# Winging of Scapula due to a Sinister Etiology

**DOI:** 10.1155/2020/8816486

**Published:** 2020-11-04

**Authors:** Shania Niromi Gunasekera, Priyanka Yogananda, Harindra Karunatilaka, Bimsara Senanayake

**Affiliations:** National Hospital of Sri Lanka, Colombo, Sri Lanka

## Abstract

**Background:**

Scapular winging is a rare but disabling deformity, which is commonly caused by lesions of the long thoracic and spinal accessory nerves that innervate the serratus anterior and trapezius muscles, respectively. Across the literature, traumatic injury to the nerves account for the majority of cases. Less common, nontraumatic causes include viral illness, neuroinflammatory conditions, toxins, compressive lesions, and C7 radiculopathy. We present a case where an apical lung malignancy causes winging of scapula by infiltrating C5–C7 roots of brachial plexus, which has been reported only once in the literature.

**Case:**

A 54-year-old male presented with recent onset painful difficulty in raising his right arm. He had no respiratory or constitutional symptoms. On examination, winging of scapula on the right side was noted with wasting and fasciculation involving the ipsilateral shoulder girdle. Proximal muscle power of the right upper limb was of 3/5 with preserved distal muscle power. No sensory loss was noted. A patch of bronchial breathing was found in the upper zone of the right lung with multiple hard cervical lymphadenopathies. Chest X-ray and contrast-enhanced computerized tomography-chest revealed a large tumor in the upper lobe of the right lung, which was confirmed to be a carcinoma of the lung. Electromyogram revealed large motor unit potentials and poor activation of right serratus anterior and internal scapulae muscles, while nerve conduction studies concluded the presence of a compressive lesion involving C5–C7 nerve roots of brachial plexus. Histology of a biopsy of the cervical lymph node confirmed metastasis from a poorly differentiated adenocarcinoma of the lung. The patient denied further investigation with MRI cervical spine. He was transferred to the cancer institute for further treatment.

**Conclusion:**

This case highlights the value of considering a compressive lung pathology with infiltration in the differential diagnosis, when evaluating winging of scapula.

## 1. Introduction

Scapular winging is a disabling deformity caused by lesions of the long thoracic and spinal accessory nerves that innervate the serratus anterior and trapezius muscles, respectively [1]. Rarely, it can be caused by damage to the dorsal scapular nerve which innervates the rhomboid muscle [1]. The spinal accessory nerve is the XI^th^ cranial nerve, while the long thoracic and dorsal scapular nerves originate from the cervical nerve roots, C5, 6, and 7, and C4, 5, and 6, respectively. Traumatic damage to these nerves causing neuropraxia accounts for the majority of the cases of scapular winging [2].

Apical lung tumors produce various neurological manifestations owing to its local infiltration or paraneoplastic effects. Reported cases of winging of scapula as the initial mode of presentation of an apical lung malignancy are extremely rare [3]. We present a case where an apical lung malignancy is manifested as winging of the ipsilateral scapula, by the infiltration of C5–C7 roots of the brachial plexus.

## 2. Case

A 54-year-old man presented with gradual onset slowly progressive weakness of the right arm over a period of 2 months. Weakness was preceded by pain in the right shoulder region which radiated down the right arm. Pain was present even at rest. Weakness was predominant in the proximal muscles of the right upper limb, with preserved fine movements of the right hand. He denied a sensory loss involving the affected limb. His left upper limb and both the lower limbs were normal with regard to the power and sensation. He did not have dysphagia, dysarthria, diplopia, or unsteadiness. His higher functions were intact. He did not report any fever, loss of appetite or weight, chronic cough, or hemoptysis. There was no recent history of trauma. He was not a smoker and neither did he consume any illicit drugs.

**Figure 1:**
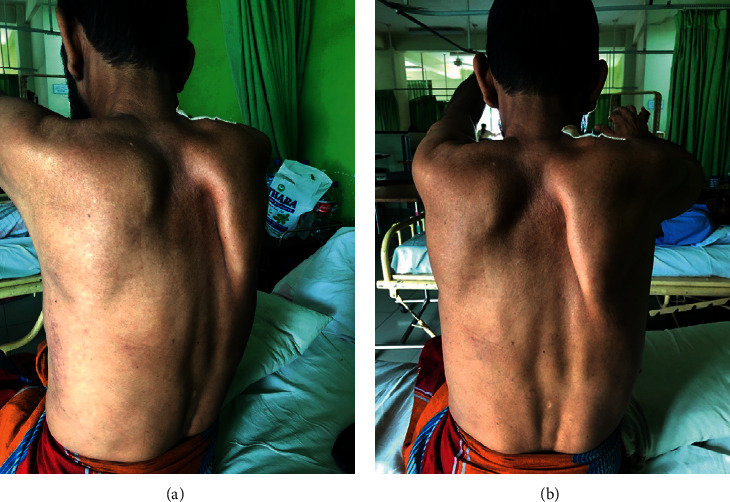
Winging of scapula. (a) Winging of the right scapula posteriorly (lateral view). (b) Winging of the right scapula (posterior view).

On examination, enlarged, multiple, hard, and fixed lymph nodes were noted in right supraclavicular fossa and anterior triangle of the neck. There was no finger clubbing. Wasting and fasciculation were observed in the muscles of the shoulder girdle and proximal part of the right upper limb, sparing forearm, and the hand muscles. Proximal muscle power was of 3/5 with preserved distal power. Posterior winging of the right scapula was noted, which became more prominent up on pushing against resistance (Figures 1(a) and 1(b)). Rest of the examination including voice, cranial nerves, pupils, contralateral upper limb, and lower limbs was normal. Bronchial breathing was heard in the upper zone of the right lung, with trachea slightly deviated to the opposite side.

**Figure 2:**
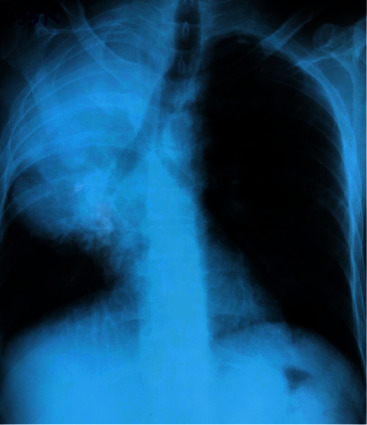
Chest radiograph—posterior-anterior view—right side apical ill-defined mass causing tracheal deviation to opposite side.

The electromyography (EMG) revealed large motor unit potentials with increasing amplitude and duration and poor activation of right serratus anterior and internal scapulae muscles. The nerve conduction studies (NCS) suggested a compressive lesion involving C5–C7 nerve roots of the brachial plexus. The chest radiograph revealed a large homogenous opacity involving the right apical region (Figure 2).

**Figure 3:**
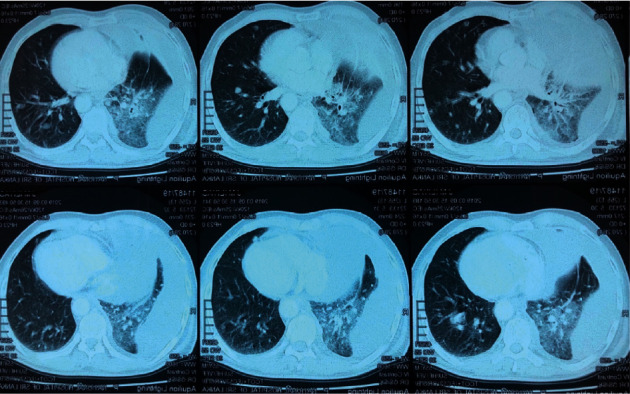
CECT chest—ill-defined enhancing malignant lesion—obliterating the right upper bronchus, right upper lobe, pulmonary artery, and pleura.

**Figure 4:**
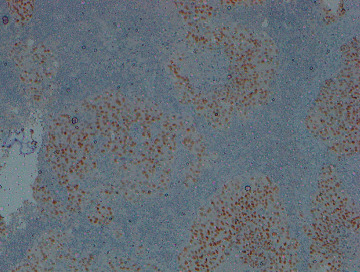
Immunohistochemistry of lymph node biopsy is positive for thyroid transcription factor 1 (TTF-1) confirming metastasis from an adenocarcinoma of lung.

Subsequent contrast-enhanced computerized tomography (CECT) showed an ill-defined enhancing malignant lesion, obliterating the right upper bronchus, right upper lobe, pulmonary artery, and pleura (Figure 3). Probable stage was IV (T4N3M1a). Enlarged necrotic lymph nodes were seen in the mediastinum, with some in the right supraclavicular fossa. Histology and immunohistochemistry (Figure 4) of a cervical lymph node revealed metastatic deposits from a poorly differentiated carcinoma, most probably of pulmonary origin.

**Figure 5:**
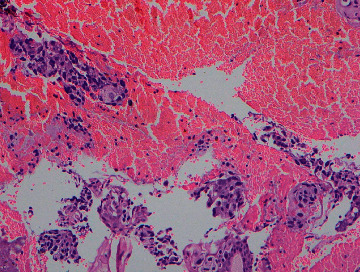
Histology of the biopsy taken from—endobronchial tumor at bronchoscopy—atypical cell clusters with oval and polygonal cells mixed with blood. Cells contain enlarged, hyperchromatic, pleomorphic nuclei and eosinophilic cytoplasm, and prominent nucleoli, compatible with adenocarcinoma of lung (H&E stain ×100).

At bronchoscopy, right upper bronchus was seen to be completely obstructed by a tumor with endobronchial invasion. The histology (Figure 5) and immunohistochemistry from the tissue obtained by bronchoscopy confirmed a poorly differentiated adenocarcinoma of the lung. Magnetic resonant imaging (MRI) of cervical spine was planned, but the patient refused to undergo further investigation.

## 3. Discussion

Scapular winging is a disabling condition that hinders the ability to lift, pull, and push against objects. Winging of scapula results from paralysis of the muscles that keeps the scapula anchored to the chest wall. The commonest cause of scapular winging is isolated serratus anterior muscle (SAM) paralysis [2]. Long thoracic nerve (LTN) innervates the serratus anterior muscle. Due to its lengthy course and superficial location, LTN is susceptible to blunt and penetrating trauma [3]. The causes of nontraumatic injury to the LTN include viral infections such as poliomyelitis, drugs, exposure to toxins (herbicides and tetanus antitoxin), muscular dystrophies (fascio-scapulo-humeral dystrophy), C7 radiculopathy, and aortic coarctation [1]. Iatrogenic causes such as thoracotomies for lung or cardiac surgeries, hematomas secondary to anticoagulation therapy, are also identified as causes of SAM palsy [3].

An apical lung malignancy causing scapular winging has been reported only once in the literature [3]. In the case reported by Toshkezi et al., apart from the winging scapula, the patient also had Horner's syndrome, which is a recognized neurological manifestation of Pancoast tumors. This would have been a clue to suspect an infiltrative pathology as the cause for the LTN palsy. But ours is a case where an apical lung malignancy caused ipsilateral winging of scapula, in the absence of any other typical neurological manifestations of a Pancoast tumor, such as hoarse voice and Horner's syndrome, which are due to the involvement of recurrent laryngeal nerve and sympathetic trunk, respectively. Neither did he have any signs of compression or infiltration of the middle and lower trunks of the brachial plexus, such as pain irradiating to the upper limb or small muscle wasting of the hand [4].

The respiratory symptoms such as cough or hemoptysis are uncommon at the initial stages of apical lung neoplasms due to its peripheral location [4]. Apart from bronchial breathing heard at auscultation and cervical lymphadenopathy, a lung malignancy was not suspected as a cause for winging of scapula, at the initial evaluation. Apical tumors can easily be missed on plain radiographs in the early course of the disease, as they might be hidden behind the clavicle and the first rib. CT and MR images would be useful to assess the local spread to adjacent neurovascular structures and the spine. The local invasion was classically seen in squamous cell carcinoma, followed by small cell carcinoma of lung, although the current case was of an adenocarcinoma [5].

## 4. Conclusion

Winging of the scapula is commonly caused by paralysis of serratus anterior muscle (SAM), due to an injury to the long thoracic nerve (LTN) or its nerve roots.

This case highlights the value of considering a compressive lung pathology with infiltration in the differential diagnosis, when evaluating winging of the scapula.

## References

[1] Martin R. M., Fish D. E. (2007). Scapular winging: anatomical review, diagnosis, and treatments. *Current Reviews in Musculoskeletal Medicine*.

[2] Gooding B. W., Geoghegan J. M., Wallace W. A., Manning P. A. (2013). Scapular winging. *Shoulder Elbow*.

[3] Toshkezi G., Dejesus J., Jabre J. F., Hohler A., Davies K. (2009). Long thoracic neuropathy caused by an apical pulmonary tumor: case report. *Journal of Neurosurgery*.

[4] Panagopoulos N., Leivaditis V., Koletsis E. (2014). Pancoast tumors: characteristics and preoperative assessment. *Journal of Thoracic Disease*.

[5] Ghaffarpour M., Firouzbakhsh S., Omrani H. G., Mansoorian B. (2002). Neurologic manifestations as the presenting symptoms in lung cancer. *Acta Medica Iranica*.

